# Essential Amino Acids (EAA) Mixture Supplementation: Effects of an Acute Administration Protocol on Myoelectric Manifestations of Fatigue in the Biceps Brachii After Resistance Exercise

**DOI:** 10.3389/fphys.2018.01140

**Published:** 2018-08-17

**Authors:** Massimo Negro, Valentina Segreto, Marco Barbero, Corrado Cescon, Luca Castelli, Luca Calanni, Giuseppe D’Antona

**Affiliations:** ^1^CRIAMS-Sport Medicine Centre, University of Pavia, Voghera, Italy; ^2^Rehabilitation Research Laboratory (2rLab), Department of Business Economics, Health and Social Care, University of Applied Sciences and Arts of Southern Switzerland, Manno, Switzerland

**Keywords:** essential amino acids, amino acids supplementation, muscle fatigue, resistance exercise, time to task, maximal voluntary contraction

## Abstract

**Background:** The purpose of this study was to investigate the acute effects of a single oral administration of an essential amino acids enriched mixture (EAA) on myoelectric descriptors of fatigue and maximal force production after a resistance exercise protocol (REP).

**Methods:** Twenty adult males (age: 27 ± 6 years; body mass: 72.7 ± 7.50 kg; height: 1.76 ± 0.06 m) were enrolled in a double-blind crossover placebo-controlled study. Subjects were randomized to receive EAA mix (0.15 g/kg BM) or a placebo (PLA) in two successive trials 7 days apart. In both trials subjects completed a REP 2 h after the ingestion of the EAA mix or PLA. Before ingestion and after REP subjects performed isometric contractions of the dominant upper limb with the elbow joint at 120 degrees: (1) two maximal voluntary contractions (MVCs) for 2–3 s; (2) at 20% MVC for 90 s; (3) at 60% MVC until exhaustion. Mean values of MVC, conduction velocity initial values (CV), fractal dimension initial values (FD), their rates of change (CV slopes, FD slopes) and the Time to perform the Task (TtT) were obtained from a multichannel surface electromyography (sEMG) recording technique. Basal blood lactate (BL) and BL after REP were measured.

**Results:** Following REP a significant decrease of MVC was observed in PLA (*P* < 0.05), while no statistical differences were found in EAA between pre-REP and post-REP. After REP, although a significant increase in BL was found in both groups (*P* < 0.0001) a higher BL Δ% was observed in PLA compared to EAA (*P* < 0.05). After REP, at 60% MVC a significant increase of CV rate of change (*P* < 0.05) was observed in PLA but not in EAA. At the same force level TtT was longer in EAA compared to PLA, with a significant TtT Δ% between groups (*P* < 0.001).

**Conclusion:** Acute EAA enriched mix administration may prevent the loss of force-generating capacity during MVC following a REP. During isometric contraction at 60% MVC after REP the EAA mix may maintain CV rate of change values with a delay in the TtT failure.

## Introduction

The possibility that a nutritional supply could have an acute effect on resistance exercise (RE) performance has attracted a great deal of interest in recent decades, and several studies on this topic have been published (Kreider et al., 2010; Smith et al., 2010; Martinez et al., 2016). The market largely claims that amino acids (AA) consumption before RE may delay the appearance of fatigue and improve physical performance. Unfortunately, this interesting hypothesis is not adequately supported in literature which primarily deals with supplements containing rapid ATP replacement aids to increase strength and power (e.g., creatine) (Butts et al., 2018), stimulants able to increase muscle activation (e.g., caffeine) (Goldstein et al., 2010), or physiological buffers known to reduce lactate production (e.g., beta-alanine) (Hoffman et al., 2018).

Authors theorized that AA, mainly branched chain amino acids (BCAAs) and essential amino acids (EAAs), can increase exercise performance through various mechanisms, such as by improving secretion of anabolic hormones, modifying fuel use during exercise, preventing mental fatigue or stimulating muscle protein synthesis/hypertrophy responses (Williams, 2005; Blomstrand, 2006; Stepto et al., 2011; Jäger et al., 2017). However, a limited number of studies is available on the effects of these AA on central and peripheral aspects of fatigue during or after RE and, therefore, this subject has yet to be adequately addressed. References available indicate a possible role of BCAA in delaying central fatigue during sustained exercise through the regulation of brain’s uptake of tryptophan, as it was historically proposed by Blomstrand (2006); more recently, Gee and Deniel (2016) hypothesized that BCAA consumption before and after intense RE may lead to a reduction of subsequent damage-induced power loss due to increased muscle recovery; Eaton et al. (2016), based on prior reference using EAA to manipulate neurotransmitters (Pardridge, 1998), evaluated the possible effects of an acute EAA administration on central fatigue and muscle activation during high-intensity exercise protocol. Data on the possible effects of acute administration of BCAA or EAA on myoelectric descriptors of fatigue (peripheral and/or central) (De Luca, 1984; Enoka and Duchateau, 2008; Mesin et al., 2009; Gonzalez-Izal et al., 2012) measured before and after a RE bout are completely lacking and research in this direction needs further advancement.

Detection of central and peripheral components of fatigue (Bigland-Ritchie et al., 1978; Kent-Braun, 1999; Schillings et al., 2003) and their relative contribution during exercise are not particularly suitable for quantification (Merletti et al., 2004) and this explains why measurement of exertion in acute AAs administration protocols is mainly based on perceived fatigue scales, cognitive functions or specific reaction time tests (Blomstrand, 2006; Greer et al., 2011; Mikulski et al., 2015; Chen et al., 2016). However, accurate and continuous recording of local muscle fatigue during tasks is possible by evaluating myoelectric activity of a selected muscle by surface electromyography (sEMG). sEMG can analyze the myoelectric manifestations of fatigue, mainly linked to two physiological exercise-related phenomena: (1) the slowing of motor unit action potentials (MUAPs) as they travel along muscle fibers, that is the reduction of their conduction velocity (CV) (Komi and Tesch, 1979; Bouissou et al., 1989; Brody et al., 1991), and (2) the synchronization of motor unit (MU) by the central nervous system, which is described as a higher occurrence of simultaneous discharge of action potentials from various MUs to increase the force production when the whole MU pool is recruited (Holtermann et al., 2009), as observed in trained subjects (Semmler, 2002) and in presence of central lesions (Datta et al., 1991). Therefore, to assess peripheral components of fatigue, CV rate of change (i.e., slope) might be measured during isometric muscular tasks (Linssen et al., 1993; Bilodeau et al., 1994; Ng and Richardson, 1996; Merletti et al., 1998; Kollmitzer et al., 1999; Dedering et al., 2000; Rainoldi et al., 2001; Gonzalez-Izal et al., 2012), whereas to evaluate central components of fatigue, sEMG descriptors of MU synchronization may be used. An sEMG descriptor of the MU synchronization level is fractal dimension (FD), which shows high reliance on MU synchronization (Troiano et al., 2008; Mesin et al., 2009).

Based on the above, the purpose of the present trial was to evaluate the acute effects of a EAA rich mixture administration on force-generating capacity during maximal voluntary contraction (MVC) and peripheral and central myoelectric manifestations of fatigue (CV and FD rates of change, respectively) in the biceps brachii muscle, measured by multichannel sEMG after a RE fatiguing protocol.

## Materials and Methods

### Subjects

Twenty recreationally active males (age: 27 ± 6 years; body mass: 72.7 ± 7.50 kg; height: 1.76 ± 0.06 m) were recruited and completed the study. The recruitment phase took place within the month before the trial. Subjects were fully informed of the aims, risks, and discomfort associated with the investigation and provided their written informed consent to participate in this study, before completing a questionnaire to assess their health status. Inclusion criteria included physiological body weight and composition (adequate for sex, age, and physical activity level) and experience in RE training. Exclusion criteria included the presence of a medical condition (musculoskeletal, respiratory, or cardiac), more than 4 days per week of physical activity and the use of nutritional supplements in the month before the trial. The study was settled at the Sport Medicine Centre, University of Pavia, Voghera, Italy and it was approved by the local ethics committee of the Swiss Italian Health and Sociality Department, Switzerland. During tests the facility presented optimal conditions of temperature and humidity, 22°C and 50% respectively.

### Study Design

This study was a double-blind randomized crossover placebo-controlled trial. Subjects received a EAA mix or an isocaloric placebo (PLA) in two successive trials separated by 7 days of washout.

### Body Composition Assessment

Anthropometric characteristics were assessed during the recruitment phase and the day before the beginning of the first trial. The day before the trial, body weight and composition were re-assessed in order to verify that the anthropometric characteristics of all the participants had been maintained 24 h before the beginning of the procedures and to potentially exclude subjects that showed significant variations compared to the values measured during the recruitment phase. Body mass and height were measured using a mechanical scale with stadiometer (Seca Ltd., Hamburg, Germany) to the nearest 0.01 kg and 0.1 m, respectively. Body mass index (BMI) was calculated as body mass (kg) divided by stature (m) squared. Fat-free mass and fat mass were measured using a bioelectric impedance analysis (BIA EFG, Akern, Florence, Italy). Measurements were carried out in the fasted state within 30 min after urine voiding. Subjects lay in supine position with limbs extended away from the trunk and wore only light clothing but no shoes or socks. Active electrodes (BIATRODES, Akern, Italy) were placed on the right side on conventional metacarpal and metatarsal lines, whereas recording electrodes were placed in standard positions at the right wrist and ankle. The physical characteristics of the subjects are illustrated in **Table 1**.

**Table 1:** Physical characteristics of subjects (*n* = 20).

Anthropometrics	Mean ±*SD*	Range
Age (yrs)	27 ± 6	21.0–39.0
BM (kg)	72.7 ± 7.50	58.0–82.1
Height (m)	1.76 ± 0.06	1.65–1.85
BMI (kg⋅m^-2^)	23.4 ± 2.03	20.1–25.2
FM (kg)	12.2 ± 3.11	8.10–19.4
FFM (kg)	56.5 ± 3.29	51.5–62.7

*Values are mean ± SD. BM, body mass; BMI, body mass index; FM, fat mass; FFM, fat free mass.*

### Experimental Procedures

**FIGURE 1:**
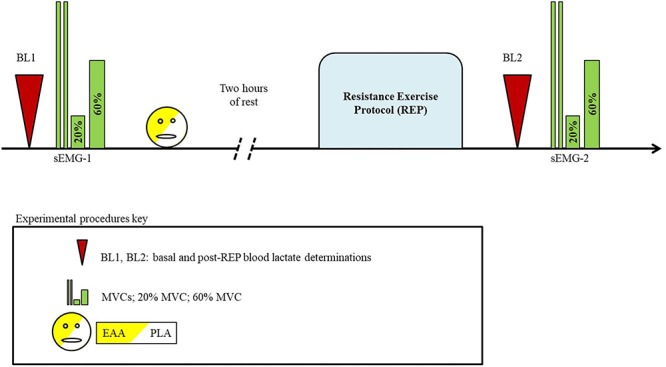
Schematic illustration of experimental procedures.

In both trials subjects refrained from strenuous physical activity and were discouraged from drinking alcohol, coffee, and beverages containing caffeine in the previous 24 h. The day of the experiment, 2 h before their arrival at the laboratory (fixed at 9:00 am), subjects had a standardized breakfast (rusks with jam; decaffeinated tea or juice). The trial consisted of the following steps: (1) basal blood lactate (BL1) determination; (2) first surface electromyography (sEMG-1) recording; (3) EAA or PLA ingestion; (4) after 2 h rest, a resistance exercise protocol (REP) was made; (5) post-REP blood lactate (BL2) determination; (6) second surface electromyography (sEMG-2) recording (**Figure 1**). An accurate simulation of the experimental procedures was performed 1 week before the first trial. This pre-trial session was conducted to allow the volunteers to familiarize themselves with all the procedures and to avoid an impairment of results caused by a “learning effect.”

### EAA Mix Administration Protocol

Participants ingested either a EAA mix, 0.15 g EAA powder/kg BM (Aminotrofic, Errekappa Euroterapici S.p.A., Milan, Italy), or a PLA (Aspartame based artificial sweetener powder with the same characteristics of appearance and flavor) both dissolved in 250 ml of water. EAA mix and PLA were administrated using an unmarked, coated sports drink bottle. Each administration was conducted in double blind fashion. Considering that a specific EAA dosage for improvement of fatigue is currently missing, we based our choice on the recent recommendations for EAA intake in athletes released by the International Society of Sports Nutrition (Jäger et al., 2017; Kerksick et al., 2017). We therefore investigated whether the dosage suggested for skeletal muscle adaptation could also be effective on RE performance and amelioration of fatigue. Artificial sweetener rather than a carbohydrate-based placebo was used to prevent a rise in insulin which could have altered the energetic metabolism. The EAA mix composition is described in **Table 2**.

**Table 2:** Essential amino acids (EAA) mixture composition for a subject of 70 kg of body mass.

Amino acids formula	Composition (g)	Composition (%)
Leucine	3.30	31.4
Lysine	1.70	16.2
Isoleucine	1.64	15.6
Valine	1.64	15.6
Threonine	0.92	8.8
Cysteine	0.39	3.7
Histidine	0.39	3.7
Phenylalanine	0.26	2.5
Methionine	0.13	1.2
Tyrosine	0.08	0.8
Tryptophan	0.05	0.5

### Surface Electromyography (sEMG) Recording Procedures

**FIGURE 2:**
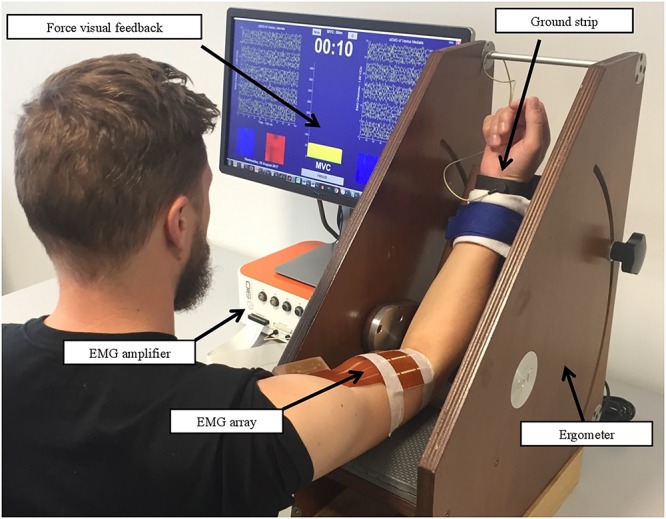
Setup of sEMG recording procedures. Written informed consent was obtained for the publication from the participant.

sEMG-1 and sEMG-2 recording procedures, before and after REP respectively, were carried out as follows: subjects’ dominant upper limb was fastened in a isometric-ergometer (MUC1, OT Bioelettronica, Turin, Italy) fitted with a load cell (CCT Transducer, linear, full scale 100 kg), in order to isolate the action of the biceps brachii. Participants were sitting, with the elbow at 120 degrees as shown in **Figure 2**.

A 64-channel bidimensional array (10 mm IED, 8 lines, 8 columns) was positioned between the distal tendon and the innervation zone of the biceps brachii, with electrode columns parallel to the orientation of the muscle fibers in order to have a pure propagation of MUAPs. Biceps brachii was selected primarily to obtain high-quality sEMG signals due to the isolation of the muscle contraction, fluency of movement, and fiber orientation. The adhesive array was applied following muscle fiber leanings in correspondence to the muscle belly previously localized by ultrasound scan (Phillips CX-30). The sEMG signals were amplified (EMG-USB2+, OT Bioelettronica, Turin, Italy) and sampled at 2048 Hz.

Following 5 min rest, two isometric MVCs were completed, separated by 2 min rest. Two contractions were performed in order to consider the highest MVC value. Participants were instructed to increase the force as maximum as they can, and to hold it as steady as possible, for 2–3 s. Participants were given verbal stimulation.

Following 2 min rest, a low intensity sustained contraction (20% MVC) was performed for 90 s.

Following 4 min rest, subjects were asked to execute a high level sustained contraction (60% MVC) until exhaustion, during which they were verbally stimulated to keep the force level as long as possible, until the force value decreased to below 5% of the target (Merletti and Roy, 1996). At 60% of MVC the time to perform the task (TtT) was registered.

### Signal Processing

Data were divided into 0.5 s periods and each variable was computed for each period. Exhaustion time was defined as the moment when force was below 5% of the target (Meduri et al., 2016). The regression line was computed for all the values from the beginning of contraction to exhaustion time. For each acquisition, the channels for the analysis were selected through visual inspection. The column showing the largest portion of propagating channels with the biggest amplitude was selected, and the channels between IZ and tendons were selected for CV computation. FD was computed for each selected channel and then averaged. FD initial value was estimated using the box counting method. Briefly, as expressed in Gitter and Czerniecki (1995), a grid of square boxes was used to cover the signal, and the number of boxes that the sEMG waveform passed through was counted. When decreasing the side of the boxes in a dichotomic process, the number of boxes required to cover the signal increased exponentially. However, by plotting the logarithm of the number of boxes counted (log N) vs. the logarithm of the inverse of the box size (log 1/S), the exponential relationship became linear. The slope of the interpolation line (estimated using the least mean squared procedure) is the FD. CV initial value (m/s) was estimated using a multichannel algorithm on double differential signals, based on the matching between signals filtered in the temporal and in the spatial domains (Farina and Merletti, 2003). CV values outside the physiological range (3–6 m/s) (Andreassen, 1987), were excluded from the analysis. CV and FD slopes were measured as rate of change (%) of CV and FD initial values.

### Resistance Exercise Protocol (REP)

Resistance exercise protocol was developed to elicit fatigue in the biceps brachii and included a Wingate exercise (warm-up + 1 × 30 s) performed on an arms ergometer (Rehab Trainer 881E, Monark, Vansbro, Sweden), with a load corresponding to 5% of BM (Dotan and Bar-Or, 1983), followed by 3 sets of biceps curling with a dumbbell (Technogym, Gambettola, Italy) until exhaustion. Dumbbell load was calculated at 70% of the 1RM and a rest of 90 s between sets was held. Total time of REP was 362 ± 12.4 s. Indirect 1RM tests to establish the dumbbell load (with the use of Brzycki’s equation) were performed 1 week before the first trial. A week was considered appropriate to exclude any individual variability in relation to the time required for a complete muscle recovery and to resolve the Delayed Onset Muscle Soreness (DOMS). All subjects experienced fatigue in completing this protocol. REP specific outcomes measured include Wingate round per minute (WRPM) and biceps curls repetitions (BCRs).

### Blood Lactate (BL) Determination

Blood sample was obtained from the earlobe and BL concentration was determined by a specific lactate detection device (Lactate Pro 2, Arkray, Kyoto, Japan).

### Statistical Analysis

Shapiro–Wilk was used to test for normal distribution of all variables prior to analysis. A paired *t*-test was used in order to compare: (1) BCRs, WRPM, BL Δ%, TtT Δ% between EAA and PLA; (2) pre-REP and post-REP outcomes in EAA e PLA: BL, MVCs, TtT at 60% of MVC, CD and FD initial values and their rates of change (slopes). Significant mean differences of absolute values were used to calculate Cohen’s *d* as an effect size. Effect sizes were considered as follows: <0.2 = trivial; 0.2 to 0.49 = small; 0.5 to 0.79 = moderate; >0.8 = large.

Data were analyzed using Prism Graphpad (San Diego, CA, United States). Statistical significance was set at *P* < 0.05. Results are presented as mean ± standard deviation (SD).

## Results

After REP a significant decrease of MVC value was observed in PLA (*P* < 0.05; effect size = 0.69), while no statistical differences were found in EAA (**Table 3**). REP outcomes showed no statistical differences for BCRs and WRPM between EAA and PLA, while statistical differences were found between BL1 (basal BL) and BL2 (post-REP) in both groups (*P* < 0.0001; EAA effect size = 3.38; PLA effect size = 4.12). A significant difference between EAA and PLA was found for BL Δ% (*P* < 0.05) (**Table 4**). Analysis of pre-REP and post-REP myoelectric manifestations of fatigue showed the following results: (1) no statistical differences in EAA and PLA for CV and FD initial values either at 20% MVC or 60% MVC; (2) at 60% MVC a significant increase of CV rate of change after REP was observed in PLA (*P* < 0.05; effect size = 0.80) but not in EAA; (3) no significant differences of FD slopes were found in either group either at 20% MVC or 60% MVC (**Table 5**).

**Table 3:** pre-REP and post-REP values of MVC in EAA and PLA.

EAA		PLA	
MVC pre-REP	19.6 ± 3.98	MVC pre-REP	19.5 ± 4.41
MVC post-REP	17.1 ± 5.27	MVC post-REP	16.3 ± 4.75^∗^

*^∗^Statistically significant change of MVC between pre-REP and post-REP in PLA (P < 0.05). Values are mean ± SD. MVC, maximal voluntary contraction (kg); REP, resistance exercise protocol.*

**Table 4:** Resistance exercise protocol (REP) outcomes in EAA and PLA.

REP Outcomes	EAA	PLA
BCRs	7 ± 2.10	8 ± 3.20
WRPM	193 ± 12.7	191 ± 20.2
BL1 (basal BL)	1.17 ± 0.29	1.14 ± 0.14
BL2 (post-REP)	4.68 ± 1.44^∗∗∗^	5.01 ± 1.32^∗∗∗^
BL Δ%	242 ± 111	349 ± 138^#^

*^∗∗∗^Statistically significant change between BL1 and BL2 in EAA and PLA (P < 0.0001). ^#^Statistically significant change of BLΔ% in PLA compared to EAA (P < 0.05). Values are mean ± SD. BCRs, biceps curls repetitions; WRPM, Wingate round per minute; BL, blood lactate concentration (mmol/L); BL Δ%, BL percentage difference between BL1 and BL2 in EAA and PLA (%).*

**Table 5:** pre-REP and post-REP values of sEMG derived myoelectric manifestations of fatigue (CV, FD initial values and their slopes).

MVC %	CV initial value pre-REP EAA	CV initial value pre-REP PLA	FD initial value pre-REP EAA	FD initial value pre-REP PLA
**20%**	4.13 ± 0.42	4.13 ± 0.68	1.67 ± 0.02	1.67 ± 0.02
**60%**	4.52 ± 0.36	4.48 ± 0.87	1.66 ± 0.03	1.68 ± 0.01

**MVC %**	**CV initial value post-REP EAA**	**CV initial value post-REP PLA**	**FD initial value post-REP EAA**	**FD initial value post-REP PLA**

**20%**	4.32 ± 0.29	4.36 ± 0.72	1.66 ± 0.02	1.66 ± 0.01
**60%**	4.87 ± 0.41	4.82 ± 0.32	1.67 ± 0.02	1.67 ± 0.01

**MVC %**	**CV slopes pre-REP EAA**	**CV slopes pre-REP PLA**	**FD slopes pre-REP EAA**	**FD slopes pre-REP PLA**

**20%**	−0.05 ± 0.04	−0.08 ± 0.09	−0.006 ± 0.009	−0.008 ± 0.006
**60%**	−0.34 ± 0.31	−0.36 ± 0.32	−0.09 ± 0.06	−0.09 ± 0.08

**MVC %**	**CV slopes post-REP EAA**	**CV slopes post-REP PLA**	**FD slopes post-REP EAA**	**FD slopes post-REP PLA**

**20%**	−0.08 ± 0.06	−0.07 ± 0.05	−0.01 ± 0.008	−0.01 ± 0.01
**60%**	−0.34 ± 0.25	−0.65 ± 0.40^∗^	−0.088 ± 0.06	−0.10 ± 0.076

*^∗^Statistically significant change of CV slopes between pre-REP and post-REP in PLA (P < 0.05). Values are Mean ± SD. CV, conduction velocity (m/s); FD, fractal dimension. CV and FD slopes (%/s).*

At 60% MVC, analysis of TtT between pre-REP and post-REP showed statistical decrease in EAA (*P* < 0.05; effect size = 0.72), but a significantly lower time was found in PLA (*P* < 0.001; effect size = 1.14). At 60% MVC, a significant difference for TtT Δ% between EAA and PLA was also found (*P* < 0.001) (**Table 6**).

**Table 6:** pre-REP and post-REP values of TtT at 60% of MVC and TtT Δ%.

EAA		PLA	
TtT pre-REP	49.4 ± 3.28	TtT pre-REP	47.4 ± 5.77
TtT post-REP	47.6 ± 1.26^∗^	TtT post-REP	40.4 ± 6.54^∗∗^
TtT Δ%	−1.27 ± 11.8	TtT Δ%	−13.9 ± 7.15^#^

*^∗^Statistically significant change between pre-REP and post-REP for TtT values in EAA (P < 0.05). ^∗∗^Statistically significant change between pre-REP and post-REP for TtT values in PLA (P < 0.001). ^#^Statistically significant change for TtT Δ% in PLA compared to EAA (P < 0.001). Values are mean ± SD. TtT, time to perform the task (s) in pre-REP and post-REP; TtT Δ%, TtT difference percentage between pre-REP and post-REP in EAA and PLA (%).*

## Discussion

### Change of Fatigue-Induced MVC

The initial aim of the present study was to examine the effects of EAA enriched mix consumption on MVC. MVC detection was used in order to sample the maximal force production during an isometric contraction and to establish how fatigue during and/or after REP could affect this muscle capacity. Our findings show that REP induced a significant decrease of MVC in PLA but not in EAA (**Table 3**). Previous works investigated the effects of acute amino acids supplementation on MVC showing positive effects (Shimomura et al., 2010; Howatson et al., 2012). However, it is important to note that these studies were designed to cause evident muscle damage rather than to evaluate acute demands in maintaining strength; in particular the capacity of nutritional intervention (BCAAs supplementation in both studies) to maintain MVC was detected several hours after RE bouts (24 h or more). As we known, strenuous RE bouts lead to a decrease of muscle function (strength and power) due to muscle damage that reaches a maximum about 24 h later and generally resolves in 48–72 h (Raastad and Hallén, 2000; Hoffman et al., 2010; Gee et al., 2012). In the studies from Shimomura et al. (2010) and Howatson et al. (2012), BCAAs supplementation showed positive effects on delayed post-exercise loss of MVC, probably due to protein synthesis stimulation, protein breakdown inhibition or both (Blomstrand et al., 2006; Zanchi et al., 2008; Nicastro et al., 2012). Conversely, in our experimental conditions, EAA mix consumption was able to reduce MVC loss measured within 15 min after REP, thus suggesting that this intervention may prevent early fatigue-induced MVC decrease.

### TtT and Myoelectric Manifestations of Fatigue

We analyzed REP-induced changes of TtT failure, as a measure of endurance, and of myoelectric descriptors as indicators of peripheral (CV) and central fatigue (FD), during submaximal isometric contractions (60% MVC) to exhaustion.

Resistance exercise protocol was able to significantly change CV slope, unlike FD slope, thus suggesting that the adopted fatiguing protocol was able to determine the appearance of peripheral fatigue, unlike changes of MU synchronization associated with central fatigue.

A lower decrease of TtT and unchanged CV rate of change due to REP was observed in EAA compared to PLA (**Tables 5, 6**). In accordance with previous findings from our lab, the maintenance of CV slopes may relate to higher resistance to peripheral arising of fatigue that has a counterpart in TtT improvement (Meduri et al., 2016).

To the best of our knowledge, these data demonstrated, for the first time, the efficacy of acute EAA mix assumption in improving sEMG myoelectric descriptors of peripheral fatigue.

In fact, so far, only two studies from the same lab (Rahmani-Nia et al., 2013; Rahmani-Nia et al., 2014) have investigated the effects of prolonged (4 weeks) L-glutamine administration on sEMG parameters in order to correlate myoelectric outcomes (median frequency, MDF, and mean power frequency, MPF) to muscle damage (Zhou et al., 2011), but no measures of fatigue descriptors were carried out.

### Potential Metabolic Role of EAA Mix Consumption

Even though EAA mix was unable to determine performance improvement in terms of number of BCRs and/or WRPM during REP, considering the effect on TtT and MVC a possible role on energy delivery during fatiguing task may be speculated. In our experimental conditions, intensity and duration of REP suggest that ATP/Creatine phosphate and glycolytic metabolisms were simultaneously involved in energy production to working muscles, as demonstrated by the observed subsequent moderate increase of blood lactate both in EAA and PLA (**Table 4**). Importantly, in EAA the increase of endurance was associated with less pronounced lactate percentage change, suggesting a potential contribution of nutritional intervention to muscle energetics, although we were not able to provide direct biochemical evidence of this assertion. At this stage, we can only underline that the role of EAA mix used in the present study in the regulation of ATP production/utilization is well established (Dioguardi, 2004; Pasini et al., 2004).

Apart from this possible mechanism, we cannot exclude that others relying on the presence of selected AA in the mixture may justify the effects here presented. In particular, as described in a previous study (Fernstrom, 2013), the ingestion of large neutral amino acids (LNAAs), notably tryptophan, tyrosine and BCAA, modifies tryptophan and tyrosine uptake into the brain and their conversion to serotonin and dopamine, respectively. This particular effect reflects the competitive nature of the transporter for LNAA at the blood–brain barrier. Typically, the administration of BCAA reduces not only tryptophan and serotonin in the brain, but also tyrosine and dopamine. This “dual effect” may have unwanted functional consequences: the positive effect on fatigue due to the serotonin reduction might be offset by the negative effects of dopamine reduction. Conversely, as reported by the same author (Fernstrom, 2013), BCAA administered in a mix with tyrosine could prevent the decline in dopamine, while still eliciting a drop in serotonin by reducing tryptophan brain uptake. However, since the concept of the ratio of serotonin to dopamine is closely related to central fatigue (Meeusen et al., 2006, 2007; Meeusen and Watson, 2007), and direct studies on the impact of this ratio on local fatigue in response to RE are lacking, it is only possible to speculate that an AA mixture containing LNAA could be effective in RE performance and improvement of fatigue based on neurotransmitters modulation.

### Limitations

This study has limitations. In particular, we were unable to determine the blood profile changes of the EAA after their ingestion. However, other authors reported that an oral EAA administration, of a similar dose to that used in the present investigation, within the 3 h before an exercise bout, is followed by a significant increase of EAA blood concentration (Eaton et al., 2016). Furthermore we can only speculate on the exact biochemical role and the contribution to the observed effects of selected amino acids in the mixture. In addition, in support to the energy role of EAA, we were only able to detect lactate percentage change, as the variation of absolute values was not significant. This was caused by the high interindividual variability of lactate increase after the REP, probably due to differences in muscular adaptation to exercise in subjects. In fact, as shown in **Table 4**, the post-REP SD values were higher compared to basal values. Notwithstanding this, we believe that the lower rise of lactate percentage in EAA (compared to PLA) should be attributed to the effect of EAA on muscle energetics. Another limitation refers to FD here used as sEMG descriptor of central fatigue. Although FD is considered a reliable index that can be used in experimental studies focusing on fatigue, literature is currently lacking of studies on validity of this parameter in MU synchronization. Other procedures, such as twitch interpolation, based on electrical stimulation of nerve trunk or branches during MVC are widely recognized in assessing central failure during voluntary activation of skeletal muscle, but compared to sEMG are slightly more uncomfortable and less usable.

## Conclusion

In order to delay fatigue and improve the overall quality of resistance training sessions, the use of pre-workout dietary supplements by both recreational and competitive athletes has been recently documented (Martinez et al., 2016; Jagim et al., 2016). This is the first study showing the efficacy of a pre-workout EAA mix administration on immediate post-RE decline of MVC, endurance capacity and peripheral muscle fatigue as evaluated with sEMG myoelectric descriptors. These data suggest that EAA-based compounds before power sport events or RE training sessions may be suitable to prevent muscle fatigue and to delay TtT failure.

## Author Contributions

MN, VS, LCas, LCal performed the experiments. MN and GD’A conceived the original idea and wrote the paper. MB and CC analyzed the data.

## Conflict of Interest Statement

The authors declare that the research was conducted in the absence of any commercial or financial relationships that could be construed as a potential conflict of interest.
